# Lipid-lowering drugs, circulating inflammatory factors, and atrial fibrillation: a mediation Mendelian randomization study

**DOI:** 10.3389/fcvm.2024.1446610

**Published:** 2024-11-05

**Authors:** Guangyang Ou, Yi Zhang, Huzhi Cai, Kunpeng Yao, Zerui Qiu, Yaowu Chen, Yang Yang, Qingyang Chen, Xinyu Chen

**Affiliations:** ^1^ The First Clinical College of Traditional Chinese Medicine, Hunan University of Chinese Medicine, Changsha, China; ^2^ Department of Urology, The First Affiliated Hospital of Jinzhou Medical University, Jinzhou Medical University, Jinzhou, China; ^3^ International Medical Department, The First Hospital of Hunan University of Chinese Medicine, Changsha, China; ^4^ Intensive Care Unit, The First Hospital of Hunan University of Chinese Medicine, Changsha, China; ^5^ Preventive Treatment Center, The First Hospital of Hunan University of Chinese Medicine, Changsha, China

**Keywords:** lipid-lowering drugs, LPL agonist, atrial fibrillation, circulating inflammatory factors, fibroblast growth factor 5, drug treatment, Mendelian randomization study

## Abstract

**Background:**

Previous studies have shown an association between lipid-lowering drugs, circulating inflammatory factors, and atrial fibrillation (AF), but the specific effects of lipid-lowering drugs on AF and whether they can be mediated by circulating inflammatory factors remain unclear.

**Methods:**

We collected 10 genetic variants encoding lipid-lowering drug targets (LDLR, HMGCR, PCSK9, NPC1L1, APOB, APOB, ABCG5, ABCG8, LPL, APOC3, and PPARA) and AF based on genome-wide association study (GWAS) summary statistics. Drug target Mendelian randomization (MR) was used to explore the causal relationship between lipid-lowering drugs and AF. In addition, we performed a mediation analysis of 91 circulating inflammatory factors to explore potential mediators. Sensitivity analyses were performed to verify the reliability of the MR Results by MR-Egger intercept test, Cochran's Q test and leave-one-out test.

**Results:**

The results of IVW method showed that LPL agonist had a protective effect on AF(OR = 0. 854, 95%CI: 0.816–0.894, *P* = 1.844E-11). However, the other nine lipid-lowering drug targets had no significant effect on AF. Notably, we found a mediator role of Fibroblast Growth Factor 5 (FGF5) in the protective effect of LPL agonist on AF with a mediator ratio of 9.22%. Sensitivity analyses supported the robustness of our findings, indicating a possible mediating pathway by which LPL agonists affect the risk of AF.

**Conclusion:**

Our study provides new insights into the complex interactions among lipid-lowering agents, circulating inflammatory factors and AF, and also identified a potential mediating role of FGF5 in the pathogenesis of AF. Our findings highlight the potential of LPL agonists and targeting specific inflammatory factors for therapeutic intervention in AF, providing promising avenues for future research and clinical strategies for the management and prevention of AF.

## 1 Introduction

Atrial Fibrillation (AF), as one of the most common arrhythmias in clinical practice, has the characteristics of high disability rate and high mortality, and can cause a series of complications such as stroke, heart failure, cognitive dysfunction, which poses a serious threat (1–4). Although many studies have identified a variety of risk factors for atrial fibrillation, including age, hypertension and cardiac structural abnormalities, the pathogenesis of AF is still not fully understood, and the efficacy and safety of existing treatments are limited.

In recent years, abnormal lipid metabolism and inflammatory response have been considered to be important factors in the pathogenesis of AF. Elevated blood levels of low-density lipoprotein cholesterol (LDL-C) and triglycerides (TG), as well as inflammatory factors such as C-reactive protein (CRP) and interleukin-6 (IL-6), are associated with an increased risk of AF (5–10). And, several studies have indicated that dyslipidemia may further lead to inflammation (11, 12). Furthermore, it has been reported in some studies that in addition to their lipid-lowering properties, various lipid-lowering drugs may also exhibit anti-inflammatory and immunomodulatory effects, effectively reducing the incidence of AF (13, 14). However, some prospective studies have presented conflicting evidence regarding the correlation (15–18). Currently, the specific relationship between lipid-lowering drugs, circulating inflammatory factors and AF is not clear, especially the mediating role of inflammatory factors in the causal relationship between lipid-lowering drugs and AF. Therefore, it is crucial to explore the effects of lipid-lowering drugs and circulating inflammatory factors on AF.

Drug-target Mendelian randomization (MR) is a new research approach specifically designed for drug repurposing, finding new therapeutic targets, and revealing the harm of drugs. Compared with the traditional observational study, MR can effectively avoid the interference of external factors and potential confounding factors, being regarded as a natural randomized controlled trial that is more cost-effective. We explored the causal relationship between LDL-C (ABCG5, ABCG8, APOB, HMGCR, LDLR, NPC1L1 and PCSK9) and TG (LPL, APOC3 and PPARA) lowering targets and AF, as well as the mediating role of circulating inflammatory factors. We hypothesize that drugs lowering LDL-C and TG levels may indirectly reduce AF risk by dampening the inflammatory response. In this way, we hope to reveal the feasibility and effectiveness of lipid-lowering drugs as potential drug targets for AF, reveal the underlying biological mechanisms, and provide more precise therapeutic strategies for the prevention and treatment of AF.

## 2 Article types

Original Research

## 3 Materials and methods

### 3.1 Study design

**Figure 1:**
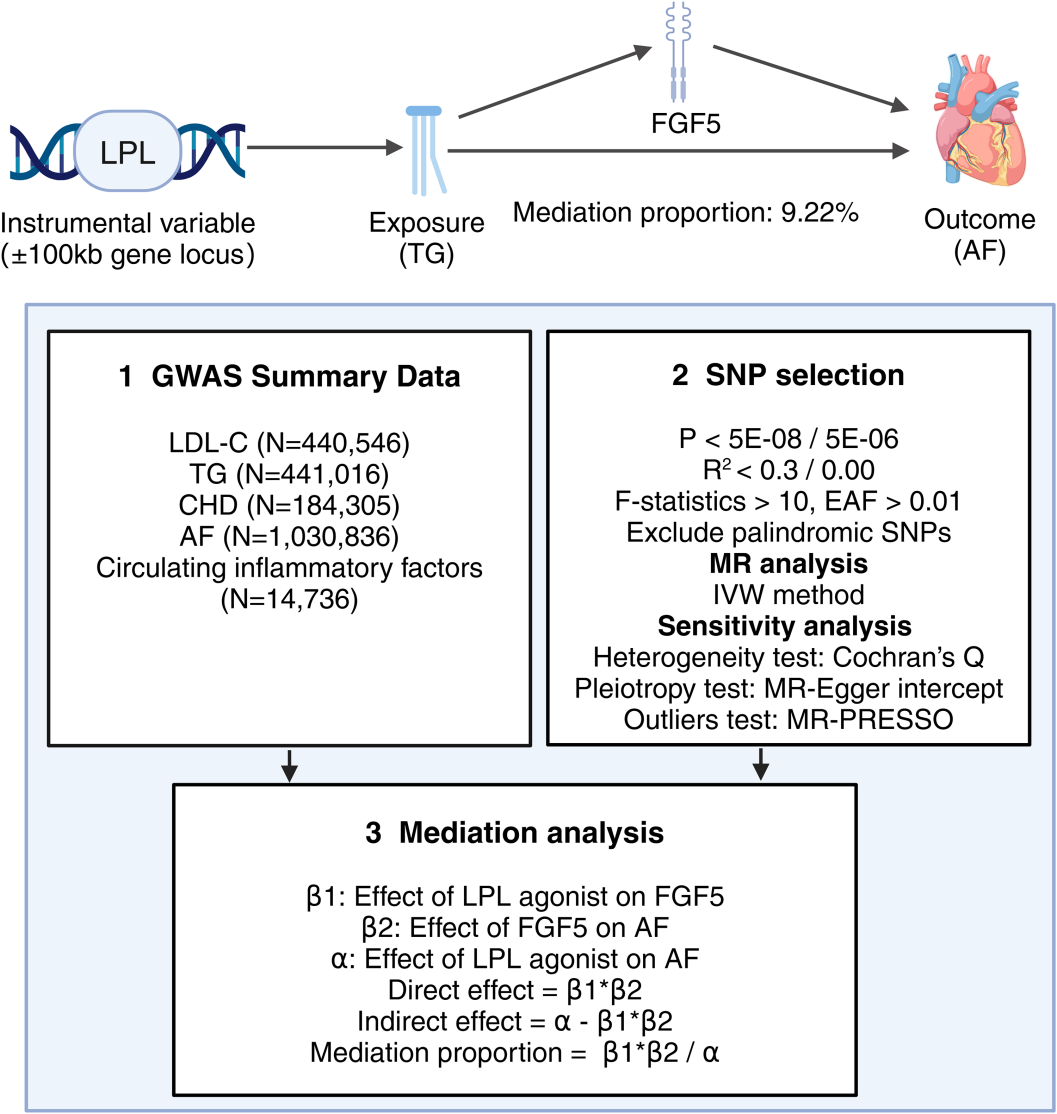
Overview of the research workflow employed in this study.

In this study, single nucleotide polymorphisms (SNPs) from the GWAS data were used as instrumental variables (IVs) for MR Analysis. The selection of SNPs for MR Analysis is based on three core assumptions (19): (1) SNPs must be strongly associated with the exposure variables; (2) SNPs should be independent of any potential confounders; and (3) SNPs influence the outcome solely through the exposure variables. This study was reported following the Strengthening the Reporting of Observational Studies in Epidemiology Using Mendelian Randomization (20, 21) (STROBE-MR). The specific process of this study is illustrated in Figure 1.

### 3.2 Genetic instruments for lipid-lowering drugs

In this study, we selected commonly used lipid-lowering drugs and the latest treatment methods, such as statins, ezetimibe, PCSK9 inhibitors, bile acid sequestrants, mipomersen, fibrates, and antisense oligonucleotides targeting apolipoprotein C-III (APOC3) mRNA. Utilizing the DrugBank database, we have identified the genes responsible for encoding the pharmacological targets of these drugs. These target genes can be further categorized into those that reduce LDL-C (such as LDLR, HMGCR, NPC1L1, PCSK9, APOB, ABCG5, and ABCG8) and those that reduce TG (including LPL, APOC3, and PPARA). LDL-C serum levels and TG serum levels are reliable downstream biomarkers of lipid-lowering drugs, so we simulated the effect of lipid-lowering drugs by obtaining SNPs within ± 100 kb of the drug target sites associated with LDL-C or TG levels to estimate the downstream effect of lipid-lowering drugs on AF risk.

Summary data on LDL-C (accession number: ieu-b-110), consisting of 440,546 Europeans and 12,321,875 SNPs, and summary data on TG (accession number: ieu-b-111), consisting of 441,016 Europeans and 12,321,875 SNPs, were obtained from the UK Biobank (UKBB). Richardson TG et al. (22) conducted a GWAS of circulating non-fasted lipoprotein lipid traits in the UKBB for low-density LDL-C, TG, and apolipoprotein B to identify lipid-associated SNPs. We first screened SNPs that were strongly associated with LDL-C or TG levels under the condition of *P* < 5E-08. Secondly, in order to ensure the independence of IVs, we excluded the interference of linkage disequilibrium (LD). For screening SNPs, R^2^ < 0.3 was used as the criterion if more than three SNPs were identified. If fewer than three SNPs were obtained, R^2^ < 0.4 was applied to ensure the MR Analysis's reliability. In addition, only SNPs with an effect allele frequency (EAF) > 0.01 were considered. Finally, in order to avoid the interference of potential confounding factors, LD trait tool was used to find and remove possible confounding factors, such as obesity, body mass index, smoking, hypertension, and diabetes (23).

### 3.3 Genetic instruments for circulating inflammatory factors

The GWAS data set for circulating inflammatory factors is available in the GWAS Catalog from a genome-wide protein quantitative trait locus study (accession number: GCST90274758 to GCST90274848). Zhao JH et al. (24) performed genome-wide pQTL mapping for 91 plasma proteins measured using the Olink Target Inflammation panel in 11 cohorts totaling 14,824 European-ancestry participants. To obtain a sufficient number of SNPs to ensure the reliability of the MR Analysis, we screened SNPs that were significantly associated with circulating inflammatory factors under the condition of *P* < 5E-06, and then excluded the interference from LD (R^2^ < 0.001, KB = 10,000) to ensure the independence of IVs.

### 3.4 Source of results

The GWAS data for AF in this study are available on the IEU open GWAS database (accession number: ebi-a-GCST006414). Nielsen JB et al. (25) tested association between 34,740,186 genetic variants and AF, comparing a total of 60,620 cases and 970,216 controls of European ancestry from six contributing studies (HUNT, deCODE, MGI, DiscovEHR, UK Biobank, and the AFGen Consortium). As lipid-lowering drugs have been approved for the treatment of coronary heart disease (CHD), CHD was selected as a positive control to validate our chosen lipid-lowering drug instrument in this study. The GWAS data for CHD were obtained from CARDIoGRAMplusC4D (accession number: ieu-a-7). Nikpay M et al. (26) assembled 60,801 cases and 123,504 controls from 48 studies for a GWAS meta-analysis of coronary artery disease. All the datasets included in this study were sourced from European populations to maintain demographic consistency.

### 3.5 Mediation analysis

First, we conducted separate assessments of the total causal effect of each of the 10 lipid-lowering drug targets on AF (*α*) and the causal effect of the lipid-lowering drug targets that were significantly associated with AF on circulating inflammatory factors (*β*1). Second, we analyzed the causal relationship between circulating inflammatory factors that were significantly associated with lipid-lowering drugs and AF (*β*2). In this MR Analysis, we excluded SNPs in the MR Analysis of lipid-lowering drug targets and AF. Finally, we utilized the coefficient product method to calculate the indirect effect (*β*1 × *β*2) and mediating proportion [(*β*1 × *β*2)/*α*] of circulating inflammatory factors on the causal relationship between lipid-lowering drugs and AF.

### 3.6 Statistical analysis

Inverse variance weighted (IVW) was used as the main analysis method, and weighted median (WME) and MR Egger's method were used for supplementary analysis. The strength of instrumental variables was evaluated using the F-statistic, with a value greater than 10 indicating no weak instrument bias (27). Heterogeneity was assessed using Cochran's Q statistics based on IVW and MR Egger methods (28). In cases of heterogeneity, the inverse variance weighted multiplicative random effects (IVW-MRE) model was employed for MR Analysis (29).MR-Egger intercept test was used to detect pleiotropy, MR-PRESSO was used to identify and remove outliers, and leave-one-out sensitivity test was used to assess whether individual SNPs had a significant effect on the overall results.

All statistical analyses were performed using R 4.3.2 and the R package (TwoSampleMR, VariantAnnotation, ieugwasr, gwasglue, and MR-PRESSO). To correct for bias caused by multiple comparisons, *P*-values were adjusted through Bonferroni correction (30).

## 4 Results

### 4.1 Positive control analysis

**Figure 2:**
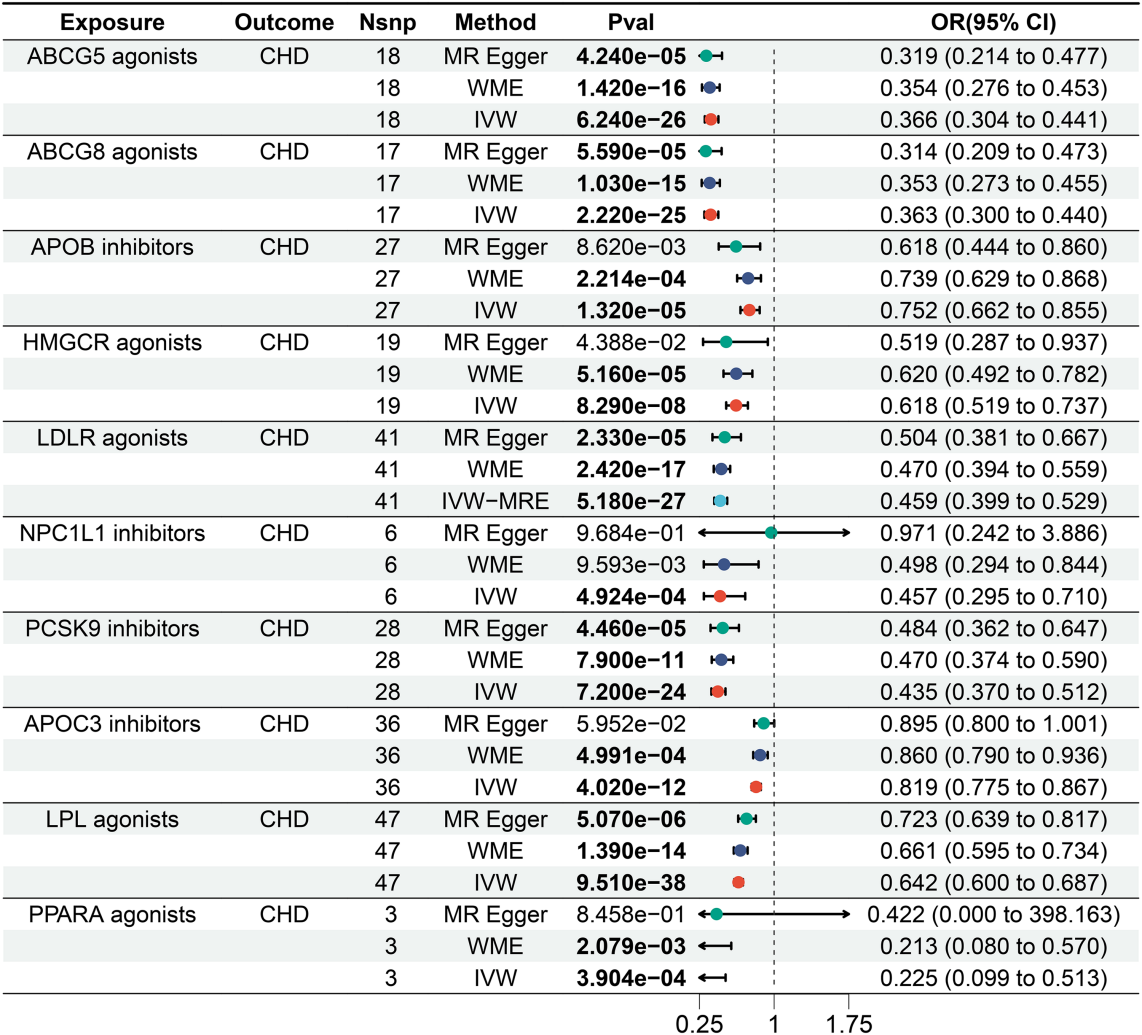
Forest plot of the results of the MR analysis between lipid-lowering drugs and coronary heart disease.

We identified causal relationships between various lipid-lowering drug targets and CHD, and as expected, the results of the IVW showed that, all 10 lipid-lowering drugs significantly reduced the risk of CHD [Bonferroni-corrected *P*-value threshold = 0.005 (0.05/10)] (Figure 2 and Supplementary Table S1).

### 4.2 MR analysis between lipid-lowering drugs and AF

**Figure 3:**
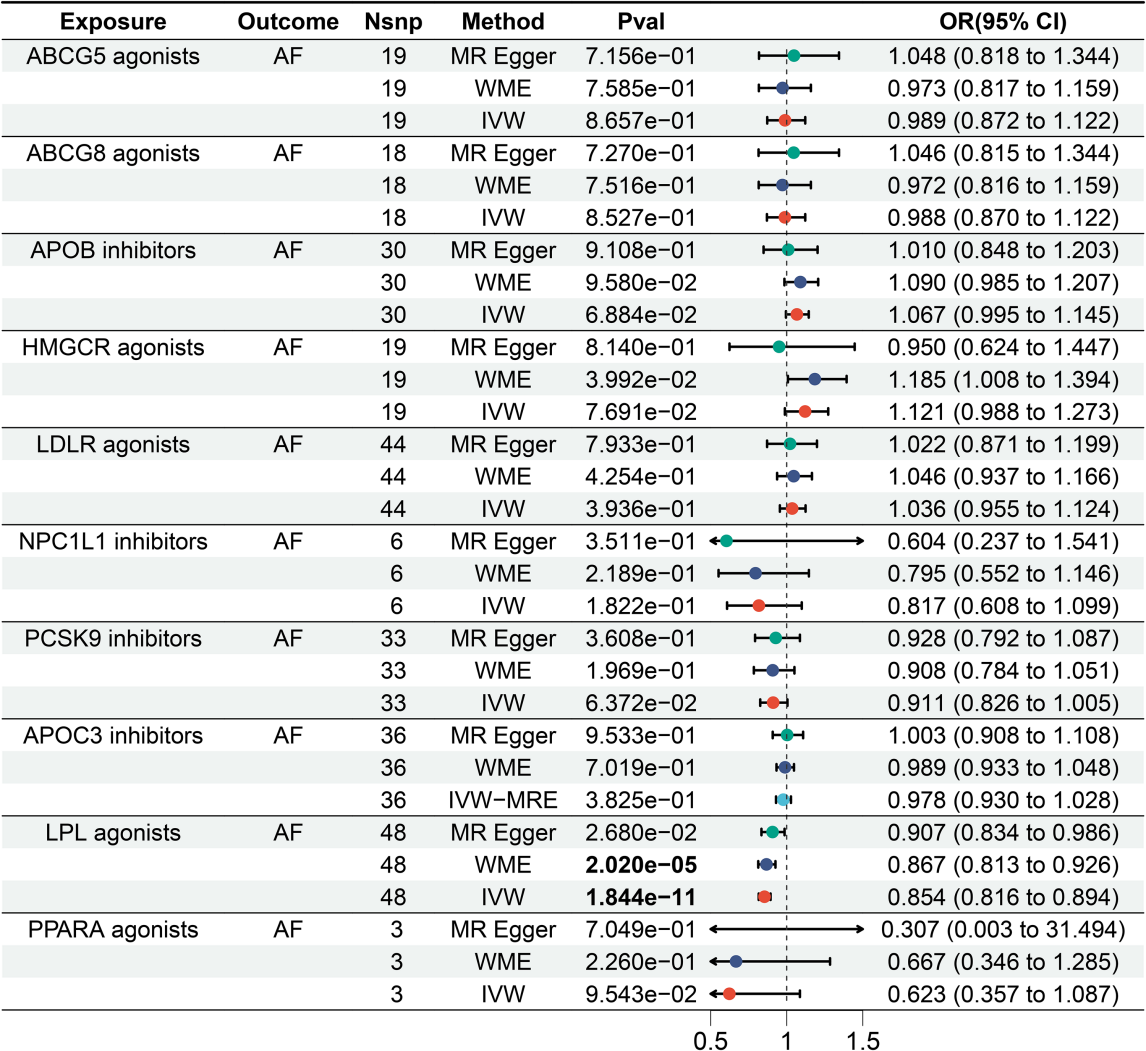
Forest plot of the results of the MR analysis between lipid-lowering drugs and atrial fibrillation.

The IVW results showed that LPL agonist had a significant effect on AF [Bonferrai-corrected *P*-value threshold = 0.005 (0.05/10)] (OR = 0. 854, 95% CI: 0.816–0.894, *P* = 1.844E-11) (Supplementary Tables S2 and S3). However, there was no significant effect of other targets on AF (Figure 3).

### 4.3 MR analysis between lipid-lowering drugs and circulating inflammatory factors

**Figure 4:**
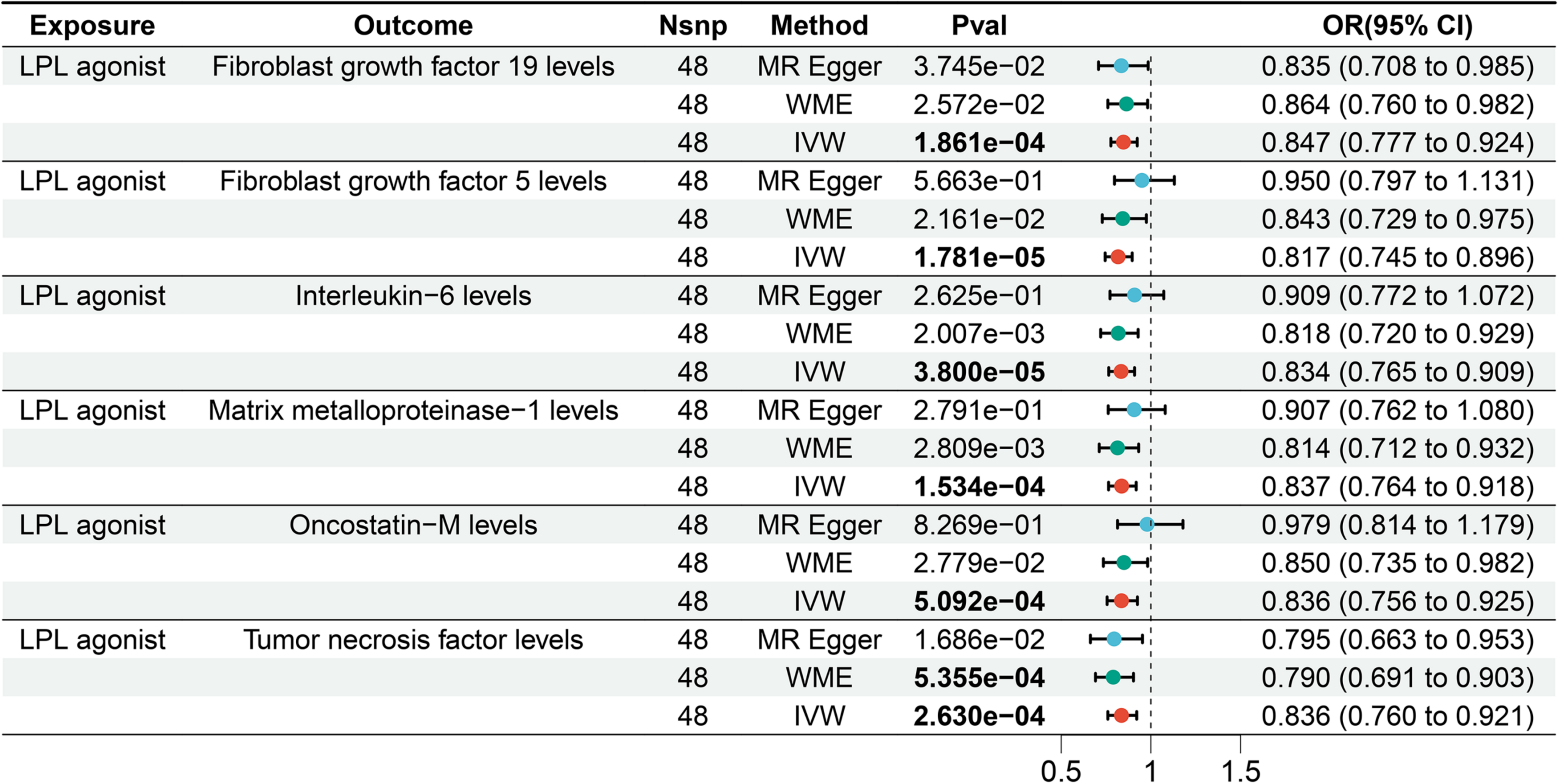
Forest plot of the results of the MR analysis between lipid-lowering drugs and circulating inflammatory factors.

We assessed the impact of LPL agonist on 91 circulating inflammatory factors. According to IVW results, we observed a causal association between LPL agonist and reduced levels of six circulating inflammatory factors after Bonferroni correction [Bonferrai-corrected *P*-value threshold = 5.495E-04 (0.05/91)] (Figure 4). Specifically, The results showed that LPL agonist was associated with decreased levels of Fibroblast growth factor (FGF) 19 (OR = 0.847, 95% CI = 0.777–0.924, *P* = 1.861E-04), FGF5 (OR = 0.817, 95% CI = 0.745–0.896, *P* = 1.781E-05), IL-6 (OR = 0.834, 95% CI = 0.765–0.909, *P* = 3.800E-05), Matrix metalloproteinase-1 (MMP-1) (OR = 0.837, 95% CI = 0.764–0.918, *P* = 1.534E-05), Oncostatin-M (OR = 0.836, 95% CI = 0.756–0.925, *P* = 5.092E-04) and Tumor necrosis factor (TNF) (OR = 0.836, 95%CI = 0.760–0.921, *P* = 2.630E-04) (Supplementary Tables S4 and 5).

### 4.4 MR analysis between circulating inflammatory factors and AF

**Figure 5:**
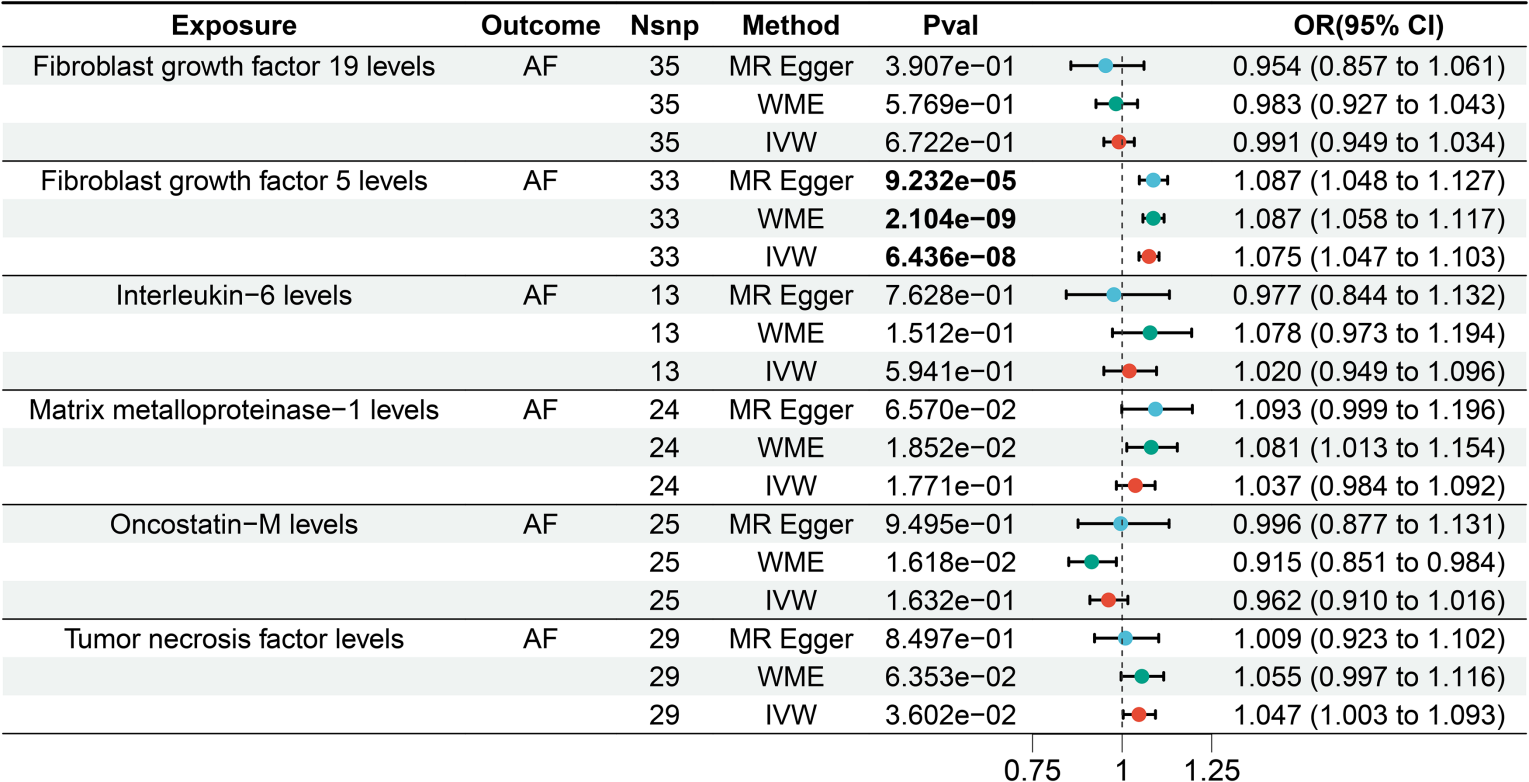
Forest plot of the results of the MR analysis between circulating inflammatory factors and atrial fibrillation.

Based on the IVW results, the MR analysis revealed a significant causal association between elevated FGF5 levels and an increased risk of AF [Bonferroni-corrected *P*-value threshold = 5.556E-03(0.05/9)] (OR = 1.075, 95% CI = 1.047–1.103, *P* = 6.436E-08) among the six LPL agonist related circulating inflammatory factors (Figure 5). However, there was no evidence of a significant causal relationship between the other 5 circulating inflammatory factors and AF.

### 4.5 Mediating role of circulating inflammatory factors in the causal relationship between lipid-lowering drugs and AF

We further investigated the mediating role of FGF5 levels in the causal relationship between LPL agonist and AF. Our findings suggest that LPL agonist may reduce the risk of AF by lowering FGF5 levels. Using the product of coefficient method, we calculated that LPL agonist is associated with a 9.22% reduction in FGF5 levels related to AF risk.

### 4.6 Results of sensitivity analysis

The MR-Egger intercept test indicated no horizontal pleiotropy in any of the results in this study. The Cochran *Q*-test showed heterogeneity in the MR Analysis between LDLR agonists and CHD, so we performed the MR Analysis using the IVW-MRE method to eliminate the bias caused by heterogeneity. In addition, an outlier (rs73015007) was identified in the MR Analysis between LDLR agonists and CHD. After removing this outlier, we repeated the MR Analysis to ensure result robustness. However, no heterogeneity or outliers were detected in the remaining MR Analyses (Supplementary Tables S7–S10). The results of leave-one-out method showed that there was no significant effect of individual SNP on the overall results in all the MR Analyses. In conclusion, the MR Results of this study are robust.

## 5 Discussion

In this study, we conducted a comprehensive exploration of the intricate relationship between LPL agonist, circulating inflammatory factors, and AF using the MR Method. The reliability of our findings was ensured through multiple sensitivity analyses and Bonferroni correction. Our study revealed a significant protective effect of LPL agonist against AF and highlighted the pivotal role of FGF5 in this process.

Through the analysis of the causal relationship between 10 lipid-lowering drug targets and AF, we discovered that LPL agonist exhibited a significant protective effect on AF. Subsequently, we conducted further investigation into the impact of LPL agonist on 91 circulating inflammatory factors. Our findings revealed that LPL agonist was significantly correlated with reduced levels of six inflammatory factors, including IL-6, TNF, and MMP-1, which was consistent with the conclusion that lipid-lowering drugs may have certain anti-inflammatory effects proposed in previous studies. LPL is an important lipid metabolic enzyme in the body, which can degrade TG in Triglyceride rich Lipoproteins (TRLs) and release free fatty acids (FFA) for the body to use (31–34). LPL agonist is a class of drugs that can reduce blood lipid levels by activating lipoprotein lipase (LPL) and accelerating the hydrolysis of TRLs.

In recent years, the association between TRLS and inflammation has been extensively studied. Moreno-Vedia et al. (35) have shown that TRL concentration and composition have a significant effect on the subclinical inflammatory state of patients with lipid metabolism disorders. Cesena et al. (36) found different patterns of association between TRL subparticles and inflammatory markers through cross-sectional analysis of the Brazilian Longitudinal Study of Adult Health (ELSA-Brasil) which supports the hypothesis that TRL may induce a low-grade inflammatory environment. A large number of previous studies have shown that inflammation plays an important role (37) in the occurrence and development of AF. Inflammation may increase the risk of AF by promoting atrial fibrosis, electrical remodeling, and causing endothelial dysfunction. Therefore, we suggest that LPL agonist may play a protective role in AF by accelerating the hydrolysis of TRLs, reducing the deposition of lipids in vascular endothelial cells, and reducing the activation and inflammatory response of endothelial cells.

However, conflicting evidence exists regarding the relationship between lipid levels and AF. A recent meta-analysis (38) found no significant correlation between TG levels and AF occurrence. This discrepancy may stem from the influence of age-related declines in TG levels or fluctuations due to dietary changes, which complicates the understanding of TG's long-term effect on AF. In contrast to simple blood lipid measurements, our study focuses on lipoprotein lipase (LPL) agonists, which dynamically regulate lipid metabolism and inflammation, offering a more comprehensive perspective on their potential protective role in AF. Therefore, while elevated TG levels may not be directly linked to AF, LPL agonists might confer protective effects through mechanisms that extend beyond lipid levels alone.

Due to the intricate nature of the LPL regulation mechanism, as well as the challenge of developing high specificity and selectivity for the target and the complexity of clinical trials, current understanding of LPL activation as the core mechanism for drug effects is still in its developmental stage (39). In this study, we found the protective effect of LPL agonist on AF using drug-target MR Method, which provides a theoretical basis for larger-scale research of LPL agonist in the future.

FGF5 is a member of the FGF family, which is mainly involved in cell proliferation, differentiation, survival and tissue repair (40). Previous studies have found that FGF5 is mainly associated with cancer and hair growth (41–44). Recent studies have indicated that FGF5 also plays a significant role in cardiovascular conditions such as hypertension and coronary artery disease (CAD) (45–48). Vatner SF et al. (49) demonstrated that FGF5 has the ability to inhibit cell apoptosis, induce cardiomyocyte hypertrophy and proliferation, ultimately leading to an increase in myocardial mass and myocardial wall thickening. Additionally, Hu et al. (50) established a causal relationship between FGF5 and Cardiac Remodeling using MR method. In addition, FGF5 also plays an important role in promoting tissue fibrosis. Hiromi H et al. (51) discovered that FGF5 was implicated in the progression of liver fibrosis and suggested that FGF5 could potentially serve as a therapeutic target for liver fibrosis in non-alcoholic steatohepatitis. In the present study, we identified a significant causal relationship between elevated levels of FGF5 and an increased AF risk. While there is no direct evidence linking FGF5 to AF, our findings suggest that this causal relationship may be attributed to the ability of FGF5 to induce cardiomyocyte hypertrophy and promote myocardial fibrosis. These effects ultimately lead to structural remodeling of the atrium, providing a potential mechanistic basis for the observed association (52, 53).

Through mediation analysis, we found that LPL agonist reduced the risk of AF by reducing the level of FGF5. The results of the mediation analysis indicated that changes in FGF5 levels accounted for 9.22% of the reduction in AF risk associated with LPL agonist treatment. This suggests that FGF5 plays a partial mediating role in the mechanism of action of LPL agonists in preventing AF. Previous studies have suggested that the protective effect of LPL agonists on AF may be linked to chronic inflammation and myocardial fibrosis, while FGF5 may increase the risk of AF through myocardial fibrosis and structural remodeling of the atrium. Therefore, it is possible that FGF5 mediates the causal relationship between LPL agonists and AF. Our study provides genetic evidence supporting the idea that FGF5 may mediate the protective effects of partial LPL agonists against AF. However, it is important to note that while our findings suggest a potential mediating association, further experimental studies are needed to confirm causality as well as to determine if other factors also play a role.

To the best of our knowledge, this is the first study to utilize MR analysis in examining the correlation between lipid-lowering drugs, circulating inflammatory factors, and AF in the general population. Our study provides new targets and strategies for the targeted treatment of AF, and points out the direction for future research. In terms of clinical implications, our findings suggest that LPL agonists could serve as novel therapeutic agents for AF prevention and management by targeting both lipid metabolism and inflammation. By reducing key inflammatory factors and influencing lipid dynamics, LPL agonists may address fundamental aspects of AF pathogenesis. Furthermore, identifying FGF5 as a mediator offers a new therapeutic target, potentially allowing for more effective interventions to prevent atrial remodeling and fibrosis associated with AF. From a research perspective, our study underscores the value of integrating genetic association studies with drug development processes. Utilizing MR analysis provides genetic evidence that supports causal relationships, aiding in the prioritization of drug targets. This approach is consistent with recent frameworks that advocate for leveraging human genetics to identify druggable targets for AF (54).

At the same time, this study also has certain limitations. First, all the data in the study were from persons of European ancestry. This reduced bias due to ethnic and regional differences but also limited the generalizability of the findings to other ethnic groups. Second, although we used a variety of sensitivity analyses in our study, potential horizontal pleiotropy may still exist. Therefore, future studies should further explore the role of LPL agonist and FGF5 in different populations and clinical settings, and verify the potential for practical application of these biomarkers in the prevention and treatment of AF.

## 6 Conclusion

In conclusion, this study supports the genetically predicted association between LPL agonist, circulating inflammatory factors, and AF. Specifically, FGF5 levels mediate the protective effect of LPL agonist against AF. These findings provide genetic evidence for the mechanism by which LPL agonists reduce the risk of AF and may inform future mechanistic and clinical studies.

## Data Availability

The datasets presented in this study can be found in online repositories. The names of the repository/repositories and accession number(s) can be found in the article/Supplementary Material.
