# Supplementary material for: Lipid-lowering drugs, circulating inflammatory factors, and atrial fibrillation: a mediation Mendelian randomization study
Source: Front Cardiovasc Med. 2024 Nov 5;11:1446610. doi: 10.3389/fcvm.2024.1446610 (PMC11573524; doi:10.3389/fcvm.2024.1446610)
Supplement: Supplementary file 1 [file Datasheet1.docx]

**Supplementary Table 1** The effect of lipid-lowering drugs on coronary heart disease

| EXPOSURE | OUTCOME | METHOD | NSNP | BETA | SE | P | OR | OR_LCI95 | OR_UCI95 |
| --- | --- | --- | --- | --- | --- | --- | --- | --- | --- |
| ABCG5 agonists | Coronary heart disease | MR Egger | 18 | -1.141 | 0.205 | 4.240E-05 | 0.319 | 0.214 | 0.477 |
| ABCG5 agonists | Coronary heart disease | Weighted median | 18 | -1.039 | 0.126 | 1.420E-16 | 0.354 | 0.276 | 0.453 |
| ABCG5 agonists | Coronary heart disease | Inverse variance weighted | 18 | -1.005 | 0.095 | 6.240E-26 | 0.366 | 0.304 | 0.441 |
| ABCG8 agonists | Coronary heart disease | MR Egger | 17 | -1.157 | 0.209 | 5.590E-05 | 0.314 | 0.209 | 0.473 |
| ABCG8 agonists | Coronary heart disease | Weighted median | 17 | -1.043 | 0.130 | 1.030E-15 | 0.353 | 0.273 | 0.455 |
| ABCG8 agonists | Coronary heart disease | Inverse variance weighted | 17 | -1.012 | 0.097 | 2.220E-25 | 0.363 | 0.300 | 0.440 |
| APOB inhibitors | Coronary heart disease | MR Egger | 27 | -0.482 | 0.169 | 8.620E-03 | 0.618 | 0.444 | 0.860 |
| APOB inhibitors | Coronary heart disease | Weighted median | 27 | -0.303 | 0.082 | 2.214E-04 | 0.739 | 0.629 | 0.868 |
| APOB inhibitors | Coronary heart disease | Inverse variance weighted | 27 | -0.284 | 0.065 | 1.320E-05 | 0.752 | 0.662 | 0.855 |
| HMGCR agonists | Coronary heart disease | MR Egger | 19 | -0.656 | 0.302 | 4.388E-02 | 0.519 | 0.287 | 0.937 |
| HMGCR agonists | Coronary heart disease | Weighted median | 19 | -0.477 | 0.118 | 5.160E-05 | 0.620 | 0.492 | 0.782 |
| HMGCR agonists | Coronary heart disease | Inverse variance weighted | 19 | -0.481 | 0.090 | 8.290E-08 | 0.618 | 0.519 | 0.737 |
| LDLR agonists | Coronary heart disease | MR Egger | 41 | -0.685 | 0.143 | 2.330E-05 | 0.504 | 0.381 | 0.667 |
| LDLR agonists | Coronary heart disease | Weighted median | 41 | -0.756 | 0.089 | 2.420E-17 | 0.470 | 0.394 | 0.559 |
| LDLR agonists | Coronary heart disease | Inverse variance weighted (multiplicative random effects) | 41 | -0.778 | 0.072 | 5.180E-27 | 0.459 | 0.399 | 0.529 |
| NPC1L1 inhibitors | Coronary heart disease | MR Egger | 6 | -0.030 | 0.708 | 9.684E-01 | 0.971 | 0.242 | 3.886 |
| NPC1L1 inhibitors | Coronary heart disease | Weighted median | 6 | -0.698 | 0.269 | 9.593E-03 | 0.498 | 0.294 | 0.844 |
| NPC1L1 inhibitors | Coronary heart disease | Inverse variance weighted | 6 | -0.782 | 0.224 | 4.924E-04 | 0.457 | 0.295 | 0.710 |
| PCSK9 inhibitors | Coronary heart disease | MR Egger | 28 | -0.726 | 0.148 | 4.460E-05 | 0.484 | 0.362 | 0.647 |
| PCSK9 inhibitors | Coronary heart disease | Weighted median | 28 | -0.755 | 0.116 | 7.900E-11 | 0.470 | 0.374 | 0.590 |
| PCSK9 inhibitors | Coronary heart disease | Inverse variance weighted | 28 | -0.831 | 0.083 | 7.200E-24 | 0.435 | 0.370 | 0.512 |
| APOC3 inhibitors | Coronary heart disease | MR Egger | 36 | -0.111 | 0.057 | 5.952E-02 | 0.895 | 0.800 | 1.001 |
| APOC3 inhibitors | Coronary heart disease | Weighted median | 36 | -0.151 | 0.043 | 4.991E-04 | 0.860 | 0.790 | 0.936 |
| APOC3 inhibitors | Coronary heart disease | Inverse variance weighted | 36 | -0.199 | 0.029 | 4.020E-12 | 0.819 | 0.775 | 0.867 |
| LPL agonists | Coronary heart disease | MR Egger | 47 | -0.325 | 0.063 | 5.070E-06 | 0.723 | 0.639 | 0.817 |
| LPL agonists | Coronary heart disease | Weighted median | 47 | -0.414 | 0.054 | 1.390E-14 | 0.661 | 0.595 | 0.734 |
| LPL agonists | Coronary heart disease | Inverse variance weighted | 47 | -0.443 | 0.035 | 9.510E-38 | 0.642 | 0.600 | 0.687 |
| PPARA agonists | Coronary heart disease | MR Egger | 3 | -0.864 | 3.495 | 8.458E-01 | 0.422 | 0.000 | 398.163 |
| PPARA agonists | Coronary heart disease | Weighted median | 3 | -1.546 | 0.502 | 2.079E-03 | 0.213 | 0.080 | 0.570 |
| PPARA agonists | Coronary heart disease | Inverse variance weighted | 3 | -1.490 | 0.420 | 3.904E-04 | 0.225 | 0.099 | 0.513 |

**Supplementary Table 2** The detail of instrumental variable of the Mendelian randomization analysis between LPL agonists and atrial fibrillation

| SNP | CHR | POS | BETA | SE | P-value | Effect alleles | Other alleles | R^2^ | F-STATISTICS |
| --- | --- | --- | --- | --- | --- | --- | --- | --- | --- |
| rs10096633 | 8 | 19830921 | -1.715E-01 | 2.994E-03 | 1.000E-200 | T | C | 6.407E-03 | 2843.877 |
| rs10102717 | 8 | 19756813 | -3.910E-02 | 2.038E-03 | 4.500E-82 | T | C | 7.294E-04 | 321.900 |
| rs113831503 | 8 | 19825055 | -3.998E-02 | 5.448E-03 | 2.200E-13 | T | C | 1.058E-04 | 46.660 |
| rs117303935 | 8 | 19837269 | -4.101E-02 | 4.514E-03 | 1.000E-19 | T | C | 1.664E-04 | 73.400 |
| rs11781692 | 8 | 19848117 | 7.577E-02 | 8.320E-03 | 8.500E-20 | A | C | 1.649E-04 | 72.719 |
| rs117910839 | 8 | 19822741 | -1.570E-01 | 5.762E-03 | 2.300E-163 | A | T | 1.499E-03 | 661.962 |
| rs117956669 | 8 | 19847645 | 5.445E-02 | 9.259E-03 | 4.100E-09 | G | T | 6.746E-05 | 29.751 |
| rs118045108 | 8 | 19913833 | 4.776E-02 | 6.542E-03 | 2.900E-13 | T | C | 1.040E-04 | 45.881 |
| rs1372343 | 8 | 19871320 | -6.519E-02 | 1.990E-03 | 1.000E-200 | T | C | 2.106E-03 | 930.540 |
| rs138295898 | 8 | 19888586 | -1.947E-01 | 5.387E-03 | 1.000E-200 | C | T | 2.703E-03 | 1195.350 |
| rs140801028 | 8 | 19731858 | -6.511E-02 | 8.730E-03 | 8.800E-14 | G | C | 1.152E-04 | 50.822 |
| rs142084074 | 8 | 19768150 | -1.491E-01 | 8.416E-03 | 3.000E-70 | A | G | 6.637E-04 | 292.875 |
| rs142565486 | 8 | 19918088 | -1.427E-01 | 7.742E-03 | 7.300E-76 | T | C | 6.709E-04 | 296.091 |
| rs144014029 | 8 | 19891915 | -7.125E-02 | 9.320E-03 | 2.100E-14 | C | T | 1.150E-04 | 50.722 |
| rs1441778 | 8 | 19727047 | 5.843E-02 | 2.725E-03 | 5.110E-102 | T | C | 8.998E-04 | 397.183 |
| rs1441779 | 8 | 19729605 | -2.600E-02 | 2.481E-03 | 1.100E-25 | C | T | 2.195E-04 | 96.803 |
| rs144469617 | 8 | 19903238 | 5.188E-02 | 7.870E-03 | 4.300E-11 | A | G | 8.589E-05 | 37.883 |
| rs147011441 | 8 | 19843748 | 4.264E-02 | 6.588E-03 | 9.700E-11 | A | G | 8.863E-05 | 39.090 |
| rs148048657 | 8 | 19774005 | 3.776E-02 | 6.666E-03 | 1.500E-08 | A | G | 7.028E-05 | 30.997 |
| rs148383135 | 8 | 19738408 | -8.750E-02 | 8.915E-03 | 9.700E-23 | A | G | 1.971E-04 | 86.945 |
| rs148754782 | 8 | 19910576 | -5.314E-02 | 6.033E-03 | 1.300E-18 | A | G | 1.532E-04 | 67.588 |
| rs17091574 | 8 | 19727569 | 3.469E-02 | 4.633E-03 | 7.000E-14 | C | T | 1.126E-04 | 49.678 |
| rs1801177 | 8 | 19805708 | 1.663E-01 | 7.587E-03 | 1.600E-106 | A | G | 9.382E-04 | 414.158 |
| rs187544997 | 8 | 19890641 | 5.272E-02 | 7.923E-03 | 2.800E-11 | G | C | 9.046E-05 | 39.898 |
| rs2044061 | 8 | 19723503 | 2.143E-02 | 2.128E-03 | 7.700E-24 | C | T | 2.002E-04 | 88.302 |
| rs2165557 | 8 | 19835050 | -4.660E-02 | 2.542E-03 | 4.700E-75 | T | A | 6.577E-04 | 290.227 |
| rs2197089 | 8 | 19826373 | -6.372E-02 | 1.988E-03 | 1.000E-200 | A | G | 2.010E-03 | 888.264 |
| rs2410622 | 8 | 19854773 | -4.708E-02 | 2.730E-03 | 1.200E-66 | C | T | 5.989E-04 | 264.268 |
| rs268 | 8 | 19813529 | 2.269E-01 | 7.395E-03 | 1.000E-200 | G | A | 1.839E-03 | 812.411 |
| rs270 | 8 | 19813676 | 2.507E-02 | 2.771E-03 | 1.500E-19 | A | C | 1.673E-04 | 73.809 |
| rs283 | 8 | 19815098 | 4.329E-02 | 2.514E-03 | 1.900E-66 | T | C | 5.980E-04 | 263.871 |
| rs287 | 8 | 19815556 | -1.169E-01 | 2.299E-03 | 1.000E-200 | G | A | 5.080E-03 | 2251.627 |
| rs308 | 8 | 19817476 | -1.594E-01 | 6.984E-03 | 2.600E-115 | G | T | 1.025E-03 | 452.627 |
| rs3289 | 8 | 19823192 | 1.542E-01 | 6.069E-03 | 2.300E-142 | C | T | 1.271E-03 | 561.306 |
| rs34761945 | 8 | 19778142 | 2.853E-02 | 3.471E-03 | 2.000E-16 | T | C | 1.382E-04 | 60.975 |
| rs3898938 | 8 | 19749390 | -3.106E-02 | 1.987E-03 | 4.500E-55 | T | C | 4.816E-04 | 212.487 |
| rs4244456 | 8 | 19710468 | 3.166E-02 | 2.010E-03 | 6.800E-56 | C | T | 4.966E-04 | 219.131 |
| rs4466415 | 8 | 19776981 | -8.699E-02 | 2.864E-03 | 1.000E-200 | C | A | 1.824E-03 | 805.689 |
| rs4557718 | 8 | 19890654 | 5.232E-02 | 2.919E-03 | 7.200E-72 | C | T | 6.275E-04 | 276.915 |
| rs4922108 | 8 | 19710388 | 1.783E-02 | 2.767E-03 | 1.200E-10 | C | T | 8.114E-05 | 35.788 |
| rs6586874 | 8 | 19717091 | 4.678E-02 | 2.448E-03 | 2.000E-81 | G | A | 7.385E-04 | 325.944 |
| rs66462329 | 8 | 19912060 | -8.073E-02 | 2.196E-03 | 1.000E-200 | A | G | 2.647E-03 | 1170.260 |
| rs73208821 | 8 | 19888313 | 4.723E-02 | 3.678E-03 | 9.600E-38 | C | G | 3.241E-04 | 142.975 |
| rs73597688 | 8 | 19755175 | -7.872E-02 | 2.543E-03 | 1.000E-200 | A | C | 1.880E-03 | 830.885 |
| rs73600043 | 8 | 19838353 | -6.524E-02 | 6.678E-03 | 1.500E-22 | T | G | 1.872E-04 | 82.588 |
| rs74444445 | 8 | 19852491 | 7.209E-02 | 6.750E-03 | 1.300E-26 | C | T | 2.423E-04 | 106.865 |
| rs75218485 | 8 | 19777695 | -1.685E-01 | 6.506E-03 | 5.500E-148 | T | C | 1.309E-03 | 577.836 |
| rs75240547 | 8 | 19757036 | -7.723E-02 | 8.748E-03 | 1.100E-18 | C | G | 1.537E-04 | 67.785 |

**Supplementary Table 3** The effect of lipid-lowering drugs on atrial fibrillation

| EXPOSURE | OUTCOME | METHOD | NSNP | BETA | SE | P | OR | OR_LCI95 | | OR_UCI95 |
| --- | --- | --- | --- | --- | --- | --- | --- | --- | --- | --- |
| ABCG5 agonists | Atrial fibrillation | MR Egger | 19 | 0.047 | 0.127 | 7.156E-01 | 1.048 | | 0.818 | 1.344 |
| ABCG5 agonists | Atrial fibrillation | Weighted median | 19 | -0.027 | 0.089 | 7.585E-01 | 0.973 | | 0.817 | 1.159 |
| ABCG5 agonists | Atrial fibrillation | Inverse variance weighted | 19 | -0.011 | 0.064 | 8.657E-01 | 0.989 | | 0.872 | 1.122 |
| ABCG8 agonists | Atrial fibrillation | MR Egger | 18 | 0.045 | 0.128 | 7.270E-01 | 1.046 | | 0.815 | 1.344 |
| ABCG8 agonists | Atrial fibrillation | Weighted median | 18 | -0.028 | 0.090 | 7.516E-01 | 0.972 | | 0.816 | 1.159 |
| ABCG8 agonists | Atrial fibrillation | Inverse variance weighted | 18 | -0.012 | 0.065 | 8.527E-01 | 0.988 | | 0.870 | 1.122 |
| APOB inhibitors | Atrial fibrillation | MR Egger | 30 | 0.010 | 0.089 | 9.108E-01 | 1.010 | | 0.848 | 1.203 |
| APOB inhibitors | Atrial fibrillation | Weighted median | 30 | 0.087 | 0.052 | 9.580E-02 | 1.090 | | 0.985 | 1.207 |
| APOB inhibitors | Atrial fibrillation | Inverse variance weighted | 30 | 0.065 | 0.036 | 6.884E-02 | 1.067 | | 0.995 | 1.145 |
| HMGCR agonists | Atrial fibrillation | MR Egger | 19 | -0.051 | 0.215 | 8.140E-01 | 0.950 | | 0.624 | 1.447 |
| HMGCR agonists | Atrial fibrillation | Weighted median | 19 | 0.170 | 0.083 | 3.992E-02 | 1.185 | | 1.008 | 1.394 |
| HMGCR agonists | Atrial fibrillation | Inverse variance weighted | 19 | 0.114 | 0.065 | 7.691E-02 | 1.121 | | 0.988 | 1.273 |
| LDLR agonists | Atrial fibrillation | MR Egger | 44 | 0.022 | 0.082 | 7.933E-01 | 1.022 | | 0.871 | 1.199 |
| LDLR agonists | Atrial fibrillation | Weighted median | 44 | 0.045 | 0.056 | 4.254E-01 | 1.046 | | 0.937 | 1.166 |
| LDLR agonists | Atrial fibrillation | Inverse variance weighted | 44 | 0.036 | 0.042 | 3.936E-01 | 1.036 | | 0.955 | 1.124 |
| NPC1L1 inhibitors | Atrial fibrillation | MR Egger | 6 | -0.503 | 0.477 | 3.511E-01 | 0.604 | | 0.237 | 1.541 |
| NPC1L1 inhibitors | Atrial fibrillation | Weighted median | 6 | -0.229 | 0.187 | 2.189E-01 | 0.795 | | 0.552 | 1.146 |
| NPC1L1 inhibitors | Atrial fibrillation | Inverse variance weighted | 6 | -0.202 | 0.151 | 1.822E-01 | 0.817 | | 0.608 | 1.099 |
| PCSK9 inhibitors | Atrial fibrillation | MR Egger | 33 | -0.075 | 0.081 | 3.608E-01 | 0.928 | | 0.792 | 1.087 |
| PCSK9 inhibitors | Atrial fibrillation | Weighted median | 33 | -0.097 | 0.075 | 1.969E-01 | 0.908 | | 0.784 | 1.051 |
| PCSK9 inhibitors | Atrial fibrillation | Inverse variance weighted | 33 | -0.093 | 0.050 | 6.372E-02 | 0.911 | | 0.826 | 1.005 |
| APOC3 inhibitors | Atrial fibrillation | MR Egger | 36 | 0.003 | 0.051 | 9.533E-01 | 1.003 | | 0.908 | 1.108 |
| APOC3 inhibitors | Atrial fibrillation | Weighted median | 36 | -0.011 | 0.030 | 7.019E-01 | 0.989 | | 0.933 | 1.048 |
| APOC3 inhibitors | Atrial fibrillation | Inverse variance weighted (multiplicative random effects) | 36 | -0.022 | 0.026 | 3.825E-01 | 0.978 | | 0.930 | 1.028 |
| LPL agonists | Atrial fibrillation | MR Egger | 48 | -0.098 | 0.043 | 2.680E-02 | 0.907 | | 0.834 | 0.986 |
| LPL agonists | Atrial fibrillation | Weighted median | 48 | -0.142 | 0.033 | 2.020E-05 | 0.867 | | 0.813 | 0.926 |
| LPL agonists | Atrial fibrillation | Inverse variance weighted | 48 | -0.158 | 0.023 | 1.844E-11 | 0.854 | | 0.816 | 0.894 |
| PPARA agonists | Atrial fibrillation | MR Egger | 3 | -1.181 | 2.363 | 7.049E-01 | 0.307 | | 0.003 | 31.494 |
| PPARA agonists | Atrial fibrillation | Weighted median | 3 | -0.405 | 0.335 | 2.260E-01 | 0.667 | | 0.346 | 1.285 |
| PPARA agonists | Atrial fibrillation | Inverse variance weighted | 3 | -0.473 | 0.284 | 9.543E-02 | 0.623 | | 0.357 | 1.087 |

**Supplementary Table 4** The detail of instrumental variable of the Mendelian randomization analyse between LPL agonists and Fibroblast growth factor 5 levels

| SNP | CHR | POS | BETA | SE | P-value | Effect alleles | Other alleles | R^2^ | F-STATISTICS |
| --- | --- | --- | --- | --- | --- | --- | --- | --- | --- |
| rs10102717 | 8 | 19756813 | -3.910E-02 | 2.038E-03 | 4.500E-82 | T | C | 7.294E-04 | 321.900 |
| rs113831503 | 8 | 19825055 | -3.998E-02 | 5.448E-03 | 2.200E-13 | T | C | 1.058E-04 | 46.660 |
| rs117303935 | 8 | 19837269 | -4.101E-02 | 4.514E-03 | 1.000E-19 | T | C | 1.664E-04 | 73.400 |
| rs11781692 | 8 | 19848117 | 7.577E-02 | 8.320E-03 | 8.500E-20 | A | C | 1.649E-04 | 72.719 |
| rs117910839 | 8 | 19822741 | -1.570E-01 | 5.762E-03 | 2.300E-163 | A | T | 1.499E-03 | 661.962 |
| rs117956669 | 8 | 19847645 | 5.445E-02 | 9.259E-03 | 4.100E-09 | G | T | 6.746E-05 | 29.751 |
| rs118045108 | 8 | 19913833 | 4.776E-02 | 6.542E-03 | 2.900E-13 | T | C | 1.040E-04 | 45.881 |
| rs1372343 | 8 | 19871320 | -6.519E-02 | 1.990E-03 | 1.000E-200 | T | C | 2.106E-03 | 930.540 |
| rs138295898 | 8 | 19888586 | -1.947E-01 | 5.387E-03 | 1.000E-200 | C | T | 2.703E-03 | 1195.350 |
| rs140801028 | 8 | 19731858 | -6.511E-02 | 8.730E-03 | 8.800E-14 | G | C | 1.152E-04 | 50.822 |
| rs142084074 | 8 | 19768150 | -1.491E-01 | 8.416E-03 | 3.000E-70 | A | G | 6.637E-04 | 292.875 |
| rs142565486 | 8 | 19918088 | -1.427E-01 | 7.742E-03 | 7.300E-76 | T | C | 6.709E-04 | 296.091 |
| rs144014029 | 8 | 19891915 | -7.125E-02 | 9.320E-03 | 2.100E-14 | C | T | 1.150E-04 | 50.722 |
| rs1441778 | 8 | 19727047 | 5.843E-02 | 2.725E-03 | 5.110E-102 | T | C | 8.998E-04 | 397.183 |
| rs1441779 | 8 | 19729605 | -2.600E-02 | 2.481E-03 | 1.100E-25 | C | T | 2.195E-04 | 96.803 |
| rs144469617 | 8 | 19903238 | 5.188E-02 | 7.870E-03 | 4.300E-11 | A | G | 8.589E-05 | 37.883 |
| rs147011441 | 8 | 19843748 | 4.264E-02 | 6.588E-03 | 9.700E-11 | A | G | 8.863E-05 | 39.090 |
| rs148048657 | 8 | 19774005 | 3.776E-02 | 6.666E-03 | 1.500E-08 | A | G | 7.028E-05 | 30.997 |
| rs148383135 | 8 | 19738408 | -8.750E-02 | 8.915E-03 | 9.700E-23 | A | G | 1.971E-04 | 86.945 |
| rs148754782 | 8 | 19910576 | -5.314E-02 | 6.033E-03 | 1.300E-18 | A | G | 1.532E-04 | 67.588 |
| rs17091574 | 8 | 19727569 | 3.469E-02 | 4.633E-03 | 7.000E-14 | C | T | 1.126E-04 | 49.678 |
| rs1801177 | 8 | 19805708 | 1.663E-01 | 7.587E-03 | 1.600E-106 | A | G | 9.382E-04 | 414.158 |
| rs187544997 | 8 | 19890641 | 5.272E-02 | 7.923E-03 | 2.800E-11 | G | C | 9.046E-05 | 39.898 |
| rs2044061 | 8 | 19723503 | 2.143E-02 | 2.128E-03 | 7.700E-24 | C | T | 2.002E-04 | 88.302 |
| rs2165557 | 8 | 19835050 | -4.660E-02 | 2.542E-03 | 4.700E-75 | T | A | 6.577E-04 | 290.227 |
| rs2197089 | 8 | 19826373 | -6.372E-02 | 1.988E-03 | 1.000E-200 | A | G | 2.010E-03 | 888.264 |
| rs2410622 | 8 | 19854773 | -4.708E-02 | 2.730E-03 | 1.200E-66 | C | T | 5.989E-04 | 264.268 |
| rs268 | 8 | 19813529 | 2.269E-01 | 7.395E-03 | 1.000E-200 | G | A | 1.839E-03 | 812.411 |
| rs270 | 8 | 19813676 | 2.507E-02 | 2.771E-03 | 1.500E-19 | A | C | 1.673E-04 | 73.809 |
| rs283 | 8 | 19815098 | 4.329E-02 | 2.514E-03 | 1.900E-66 | T | C | 5.980E-04 | 263.871 |
| rs287 | 8 | 19815556 | -1.169E-01 | 2.299E-03 | 1.000E-200 | G | A | 5.080E-03 | 2251.627 |
| rs308 | 8 | 19817476 | -1.594E-01 | 6.984E-03 | 2.600E-115 | G | T | 1.025E-03 | 452.627 |
| rs3289 | 8 | 19823192 | 1.542E-01 | 6.069E-03 | 2.300E-142 | C | T | 1.271E-03 | 561.306 |
| rs34761945 | 8 | 19778142 | 2.853E-02 | 3.471E-03 | 2.000E-16 | T | C | 1.382E-04 | 60.975 |
| rs3898938 | 8 | 19749390 | -3.106E-02 | 1.987E-03 | 4.500E-55 | T | C | 4.816E-04 | 212.487 |
| rs4244456 | 8 | 19710468 | 3.166E-02 | 2.010E-03 | 6.800E-56 | C | T | 4.966E-04 | 219.131 |
| rs4466415 | 8 | 19776981 | -8.699E-02 | 2.864E-03 | 1.000E-200 | C | A | 1.824E-03 | 805.689 |
| rs4557718 | 8 | 19890654 | 5.232E-02 | 2.919E-03 | 7.200E-72 | C | T | 6.275E-04 | 276.915 |
| rs4922108 | 8 | 19710388 | 1.783E-02 | 2.767E-03 | 1.200E-10 | C | T | 8.114E-05 | 35.788 |
| rs6586874 | 8 | 19717091 | 4.678E-02 | 2.448E-03 | 2.000E-81 | G | A | 7.385E-04 | 325.944 |
| rs66462329 | 8 | 19912060 | -8.073E-02 | 2.196E-03 | 1.000E-200 | A | G | 2.647E-03 | 1170.260 |
| rs73208821 | 8 | 19888313 | 4.723E-02 | 3.678E-03 | 9.600E-38 | C | G | 3.241E-04 | 142.975 |
| rs73597688 | 8 | 19755175 | -7.872E-02 | 2.543E-03 | 1.000E-200 | A | C | 1.880E-03 | 830.885 |
| rs73600043 | 8 | 19838353 | -6.524E-02 | 6.678E-03 | 1.500E-22 | T | G | 1.872E-04 | 82.588 |
| rs74444445 | 8 | 19852491 | 7.209E-02 | 6.750E-03 | 1.300E-26 | C | T | 2.423E-04 | 106.865 |
| rs75218485 | 8 | 19777695 | -1.685E-01 | 6.506E-03 | 5.500E-148 | T | C | 1.309E-03 | 577.836 |
| rs75240547 | 8 | 19757036 | -7.723E-02 | 8.748E-03 | 1.100E-18 | C | G | 1.537E-04 | 67.785 |
| rs77312736 | 8 | 19714837 | 2.398E-02 | 3.292E-03 | 3.200E-13 | A | G | 1.090E-04 | 48.090 |

**Supplementary Table 5** The effect of LPL agonist on circulating inflammatory factors

| EXPOSURE | OUTCOME | METHOD | NSNP | BETA | SE | P | OR | OR_LCI95 | OR_UCI95 |
| --- | --- | --- | --- | --- | --- | --- | --- | --- | --- |
| LPL agonist | Fibroblast growth factor 19 levels | MR Egger | 48 | -0.180 | 0.084 | 3.745E-02 | 0.835 | 0.708 | 0.985 |
| LPL agonist | Fibroblast growth factor 19 levels | Weighted median | 48 | -0.146 | 0.065 | 2.572E-02 | 0.864 | 0.760 | 0.982 |
| LPL agonist | Fibroblast growth factor 19 levels | Inverse variance weighted | 48 | -0.166 | 0.044 | 1.861E-04 | 0.847 | 0.777 | 0.924 |
| LPL agonist | Fibroblast growth factor 5 levels | MR Egger | 48 | -0.052 | 0.089 | 5.663E-01 | 0.950 | 0.797 | 1.131 |
| LPL agonist | Fibroblast growth factor 5 levels | Weighted median | 48 | -0.170 | 0.074 | 2.161E-02 | 0.843 | 0.729 | 0.975 |
| LPL agonist | Fibroblast growth factor 5 levels | Inverse variance weighted | 48 | -0.202 | 0.047 | 1.781E-05 | 0.817 | 0.745 | 0.896 |
| LPL agonist | Interleukin-6 levels | MR Egger | 48 | -0.095 | 0.084 | 2.625E-01 | 0.909 | 0.772 | 1.072 |
| LPL agonist | Interleukin-6 levels | Weighted median | 48 | -0.201 | 0.065 | 2.007E-03 | 0.818 | 0.720 | 0.929 |
| LPL agonist | Interleukin-6 levels | Inverse variance weighted | 48 | -0.182 | 0.044 | 3.800E-05 | 0.834 | 0.765 | 0.909 |
| LPL agonist | Matrix metalloproteinase-1 levels | MR Egger | 48 | -0.097 | 0.089 | 2.791E-01 | 0.907 | 0.762 | 1.080 |
| LPL agonist | Matrix metalloproteinase-1 levels | Weighted median | 48 | -0.205 | 0.069 | 2.809E-03 | 0.814 | 0.712 | 0.932 |
| LPL agonist | Matrix metalloproteinase-1 levels | Inverse variance weighted | 48 | -0.177 | 0.047 | 1.534E-04 | 0.837 | 0.764 | 0.918 |
| LPL agonist | Oncostatin-M levels | MR Egger | 48 | -0.021 | 0.095 | 8.269E-01 | 0.979 | 0.814 | 1.179 |
| LPL agonist | Oncostatin-M levels | Weighted median | 48 | -0.163 | 0.074 | 2.779E-02 | 0.850 | 0.735 | 0.982 |
| LPL agonist | Oncostatin-M levels | Inverse variance weighted | 48 | -0.179 | 0.051 | 5.092E-04 | 0.836 | 0.756 | 0.925 |
| LPL agonist | Tumor necrosis factor levels | MR Egger | 48 | -0.230 | 0.093 | 1.686E-02 | 0.795 | 0.663 | 0.953 |
| LPL agonist | Tumor necrosis factor levels | Weighted median | 48 | -0.236 | 0.068 | 5.355E-04 | 0.790 | 0.691 | 0.903 |
| LPL agonist | Tumor necrosis factor levels | Inverse variance weighted | 48 | -0.179 | 0.049 | 2.630E-04 | 0.836 | 0.760 | 0.921 |

**Supplementary Table 6** The effect of circulating inflammatory factors on atrial fibrillation

| EXPOSURE | OUTCOME | METHOD | NSNP | BETA | SE | P | OR | OR_LCI95 | OR_UCI95 |
| --- | --- | --- | --- | --- | --- | --- | --- | --- | --- |
| Fibroblast growth factor 19 levels | Atrial fibrillation | MR Egger | 35 | -0.047 | 0.054 | 3.907E-01 | 0.954 | 0.857 | 1.061 |
| Fibroblast growth factor 19 levels | Atrial fibrillation | Weighted median | 35 | -0.017 | 0.030 | 5.769E-01 | 0.983 | 0.927 | 1.043 |
| Fibroblast growth factor 19 levels | Atrial fibrillation | Inverse variance weighted | 35 | -0.009 | 0.022 | 6.722E-01 | 0.991 | 0.949 | 1.034 |
| Fibroblast growth factor 5 levels | Atrial fibrillation | MR Egger | 33 | 0.083 | 0.019 | 9.232E-05 | 1.087 | 1.048 | 1.127 |
| Fibroblast growth factor 5 levels | Atrial fibrillation | Weighted median | 33 | 0.083 | 0.014 | 2.104E-09 | 1.087 | 1.058 | 1.117 |
| Fibroblast growth factor 5 levels | Atrial fibrillation | Inverse variance weighted | 33 | 0.072 | 0.013 | 6.436E-08 | 1.075 | 1.047 | 1.103 |
| Interleukin-6 levels | Atrial fibrillation | MR Egger | 13 | -0.023 | 0.075 | 7.628E-01 | 0.977 | 0.844 | 1.132 |
| Interleukin-6 levels | Atrial fibrillation | Weighted median | 13 | 0.075 | 0.052 | 1.512E-01 | 1.078 | 0.973 | 1.194 |
| Interleukin-6 levels | Atrial fibrillation | Inverse variance weighted | 13 | 0.020 | 0.037 | 5.941E-01 | 1.020 | 0.949 | 1.096 |
| Matrix metalloproteinase-1 levels | Atrial fibrillation | MR Egger | 24 | 0.089 | 0.046 | 6.570E-02 | 1.093 | 0.999 | 1.196 |
| Matrix metalloproteinase-1 levels | Atrial fibrillation | Weighted median | 24 | 0.078 | 0.033 | 1.852E-02 | 1.081 | 1.013 | 1.154 |
| Matrix metalloproteinase-1 levels | Atrial fibrillation | Inverse variance weighted | 24 | 0.036 | 0.027 | 1.771E-01 | 1.037 | 0.984 | 1.092 |
| Oncostatin-M levels | Atrial fibrillation | MR Egger | 25 | -0.004 | 0.065 | 9.495E-01 | 0.996 | 0.877 | 1.131 |
| Oncostatin-M levels | Atrial fibrillation | Weighted median | 25 | -0.089 | 0.037 | 1.618E-02 | 0.915 | 0.851 | 0.984 |
| Oncostatin-M levels | Atrial fibrillation | Inverse variance weighted | 25 | -0.039 | 0.028 | 1.632E-01 | 0.962 | 0.910 | 1.016 |
| Tumor necrosis factor levels | Atrial fibrillation | MR Egger | 29 | 0.009 | 0.045 | 8.497E-01 | 1.009 | 0.923 | 1.102 |
| Tumor necrosis factor levels | Atrial fibrillation | Weighted median | 29 | 0.053 | 0.029 | 6.353E-02 | 1.055 | 0.997 | 1.116 |
| Tumor necrosis factor levels | Atrial fibrillation | Inverse variance weighted | 29 | 0.046 | 0.022 | 3.602E-02 | 1.047 | 1.003 | 1.093 |

**Supplementary Table 7** The result of heterogeneity test and horizontal pleiotropic test for MR analysis between lipid-lowering drugs and coronary heart disease

| Exposures | Heterogeneity test | | | Horizontal pleiotropic test | | |
| --- | --- | --- | --- | --- | --- | --- |
|  | Method | Q | Q_pval | Method | egger_intercept | p-value |
| ABCG5 agonists | MR Egger | 17.832 | 3.338E-01 | MR-Egger intercept | -6.412E-03 | 4.606E-01 |
|  | Inverse variance weighted | 18.469 | 3.598E-01 |  |  |  |
| ABCG8 agonists | MR Egger | 17.147 | 3.102E-01 | MR-Egger intercept | -6.766E-03 | 4.437E-01 |
|  | Inverse variance weighted | 17.854 | 3.325E-01 |  |  |  |
| APOB inhibitors | MR Egger | 35.634 | 7.734E-02 | MR-Egger intercept | -1.196E-02 | 2.183E-01 |
|  | Inverse variance weighted | 37.907 | 6.177E-02 |  |  |  |
| HMGCR agonists | MR Egger | 16.345 | 4.995E-01 | MR-Egger intercept | -8.047E-03 | 5.499E-01 |
|  | Inverse variance weighted | 16.717 | 5.426E-01 |  |  |  |
| LDLR agonists | MR Egger | 67.209 | 3.319E-03 | MR-Egger intercept | 4.644E-03 | 4.514E-01 |
|  | Inverse variance weighted | 68.207 | 3.571E-03 |  |  |  |
| NPC1L1 inhibitors | MR Egger | 0.556 | 9.678E-01 | MR-Egger intercept | 1.879E-02 | 3.251E-01 |
|  | Inverse variance weighted | 1.812 | 8.745E-01 |  |  |  |
| PCSK9 inhibitors | MR Egger | 25.777 | 4.754E-01 | MR-Egger intercept | 4.765E-03 | 4.034E-01 |
|  | Inverse variance weighted | 26.498 | 4.911E-01 |  |  |  |
| APOC3 inhibitors | MR Egger | 27.600 | 7.730E-01 | MR-Egger intercept | 9.920E-03 | 8.355E-02 |
|  | Inverse variance weighted | 30.779 | 6.721E-01 |  |  |  |
| PPARA agonists | MR Egger | 0.073 | 7.871E-01 | MR-Egger intercept | 1.298E-02 | 8.863E-01 |
|  | Inverse variance weighted | 0.106 | 9.486E-01 |  |  |  |
| LPL agonists | MR Egger | 38.674 | 7.355E-01 | MR-Egger intercept | 9.775E-03 | 2.894E-02 |
|  | Inverse variance weighted | 43.767 | 5.663E-01 |  |  |  |

**Supplementary Table 8** The result of heterogeneity test and horizontal pleiotropic test for MR analysis between lipid-lowering drugs and atrial fibrillation

| Exposures | Heterogeneity test | | | Horizontal pleiotropic test | | |
| --- | --- | --- | --- | --- | --- | --- |
|  | Method | Q | Q_pval | Method | egger_intercept | p-value |
| ABCG5 agonists | MR Egger | 14.233 | 0.651 | MR-Egger intercept | 2.651E-03 | 6.029E-01 |
|  | Inverse variance weighted | 14.514 | 0.695 |  |  |  |
| ABCG8 agonists | MR Egger | 14.220 | 0.582 | MR-Egger intercept | 2.617E-03 | 6.085E-01 |
|  | Inverse variance weighted | 14.493 | 0.632 |  |  |  |
| APOB inhibitors | MR Egger | 18.461 | 0.914 | MR-Egger intercept | -3.475E-03 | 5.071E-01 |
|  | Inverse variance weighted | 18.912 | 0.924 |  |  |  |
| HMGCR agonists | MR Egger | 13.431 | 0.707 | MR-Egger intercept | -7.369E-03 | 4.296E-01 |
|  | Inverse variance weighted | 14.086 | 0.723 |  |  |  |
| LDLR agonists | MR Egger | 57.948 | 0.052 | MR-Egger intercept | -7.103E-04 | 8.425E-01 |
|  | Inverse variance weighted | 58.003 | 0.063 |  |  |  |
| NPC1L1 inhibitors | MR Egger | 2.049 | 0.727 | MR-Egger intercept | -7.385E-03 | 5.417E-01 |
|  | Inverse variance weighted | 2.493 | 0.778 |  |  |  |
| PCSK9 inhibitors | MR Egger | 21.682 | 0.893 | MR-Egger intercept | 9.815E-04 | 7.756E-01 |
|  | Inverse variance weighted | 21.764 | 0.914 |  |  |  |
| APOC3 inhibitors | MR Egger | 204.578 | 0.013 | MR-Egger intercept | 2.114E-03 | 2.000E-01 |
|  | Inverse variance weighted | 206.669 | 0.012 |  |  |  |
| PPARA agonists | MR Egger | 0.303 | 0.582 | MR-Egger intercept | -1.468E-02 | 8.134E-01 |
|  | Inverse variance weighted | 0.394 | 0.821 |  |  |  |
| LPL agonists | MR Egger | 41.395 | 0.665 | MR-Egger intercept | 4.976E-03 | 1.033E-01 |
|  | Inverse variance weighted | 44.157 | 0.591 |  |  |  |

**Supplementary Table 9** The result of heterogeneity test and horizontal pleiotropic test for MR analysis between LPL agonists and circulating inflammatory factors

| Outcomes | Heterogeneity test | | | Horizontal pleiotropic test | | |
| --- | --- | --- | --- | --- | --- | --- |
|  | Method | Q | Q_pval | Method | egger_intercept | p-value |
| Fibroblast growth factor 19 levels | MR Egger | 34.325 | 0.898 | MR-Egger intercept | -1.116E-03 | 8.394E-01 |
|  | Inverse variance weighted | 34.366 | 0.915 |  |  |  |
| Fibroblast growth factor 5 levels | MR Egger | 27.939 | 0.984 | MR-Egger intercept | 1.160E-02 | 5.271E-02 |
|  | Inverse variance weighted | 31.894 | 0.955 |  |  |  |
| Interleukin-6 levels | MR Egger | 31.459 | 0.950 | MR-Egger intercept | 6.643E-03 | 2.282E-01 |
|  | Inverse variance weighted | 32.950 | 0.940 |  |  |  |
| Matrix metalloproteinase-1 levels | MR Egger | 50.981 | 0.284 | MR-Egger intercept | 6.139E-03 | 2.937E-01 |
|  | Inverse variance weighted | 52.231 | 0.278 |  |  |  |
| Oncostatin-M levels | MR Egger | 58.217 | 0.107 | MR-Egger intercept | 1.206E-02 | 5.590E-02 |
|  | Inverse variance weighted | 63.086 | 0.058 |  |  |  |
| Tumor necrosis factor levels | MR Egger | 21.228 | 0.999 | MR-Egger intercept | -3.944E-03 | 5.176E-01 |
|  | Inverse variance weighted | 21.654 | 0.999 |  |  |  |

**Supplementary Table 10** The result of heterogeneity test and horizontal pleiotropic test for MR analysis between circulating inflammatory factors and atrial fibrillation

| Exposures | Heterogeneity test | | | Horizontal pleiotropic test | | |
| --- | --- | --- | --- | --- | --- | --- |
|  | Method | Q | Q_pval | Method | egger_intercept | p-value |
| Fibroblast growth factor 19 levels | MR Egger | 44.137 | 0.093 | MR-Egger intercept | 4.500E-03 | 4.497E-01 |
|  | Inverse variance weighted | 44.919 | 0.100 |  |  |  |
| Fibroblast growth factor 5 levels | MR Egger | 40.678 | 0.114 | MR-Egger intercept | -2.959E-03 | 3.835E-01 |
|  | Inverse variance weighted | 41.703 | 0.117 |  |  |  |
| Interleukin-6 levels | MR Egger | 10.405 | 0.494 | MR-Egger intercept | 1.545E-02 | 9.760E-02 |
|  | Inverse variance weighted | 10.832 | 0.543 |  |  |  |
| Matrix metalloproteinase-1 levels | MR Egger | 29.933 | 0.120 | MR-Egger intercept | -1.343E-02 | 9.133E-02 |
|  | Inverse variance weighted | 32.609 | 0.088 |  |  |  |
| Oncostatin-M levels | MR Egger | 28.874 | 0.185 | MR-Egger intercept | -3.375E-03 | 5.559E-01 |
|  | Inverse variance weighted | 29.322 | 0.208 |  |  |  |
| Tumor necrosis factor levels | MR Egger | 29.854 | 0.321 | MR-Egger intercept | 4.364E-03 | 3.522E-01 |
|  | Inverse variance weighted | 30.845 | 0.324 |  |  |  |
