# Supplementary figures and images for: Lipid-lowering drugs, circulating inflammatory factors, and atrial fibrillation: a mediation Mendelian randomization study
Source: Front Cardiovasc Med. 2024 Nov 5;11:1446610. doi: 10.3389/fcvm.2024.1446610 (PMC11573524; doi:10.3389/fcvm.2024.1446610)

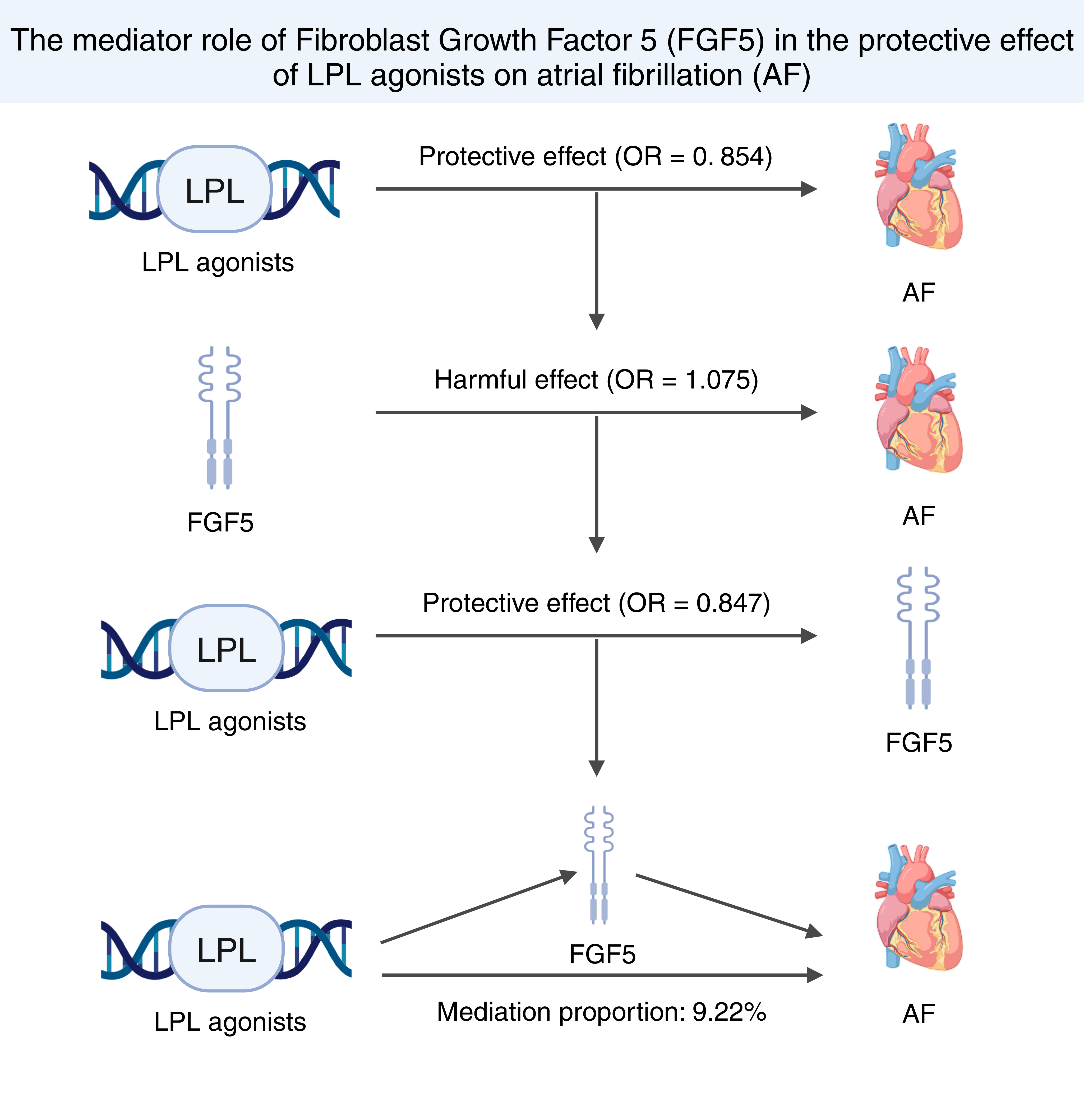

Supplement: Supplementary file 2 [file Image1.tif]
